# Estimating the density of raccoon dogs (*Nyctereutes procyonoides*) and water deer (*Hydropotes inermis*) using thermal real-time drone surveys and distance sampling

**DOI:** 10.1038/s41598-026-50722-9

**Published:** 2026-04-28

**Authors:** Yeon Woo Lee, Sumin Jeon, Jihye Son, Minyoung Kim, Hyeok Jae Lee, Soo Kyeong Hwang, Ryeoeun Youn, Choonghyun Kang, Jiwon Choi, Tae Ho Lee, Hwanhee Seo, Jong Koo Lee

**Affiliations:** Division of Life Sciences, College of Sciences and Bioengineering, Incheon National University, 119 Academy-ro, Yeonsu-gu, Incheon, 22012 Republic of Korea

**Keywords:** Wildlife drone survey, Real-time drone survey, Distance sampling, Density estimation, Raccoon dog, Water deer, Ecology, Ecology, Zoology

## Abstract

Accurate estimation of population density and spatial distribution is essential for wildlife conservation and management, yet conventional survey methods face substantial limitations. Estimating the density of medium- to large-sized mammals remains particularly challenging due to difficulties in direct observation and methodological constraints. This study qualitatively evaluated the detection performance and species identification capability of real-time drone surveys and assessed the applicability of distance sampling for density estimation. Surveys were conducted in a reedbed in Yeonsu, Incheon, and a plantation forest in Chungju, South Korea. Real-time drone surveys combined with line-transect sampling were applied in Yeonsu, whereas point-transect sampling was used in Chungju. Across nine line-transect surveys, 270 raccoon dogs (*Nyctereutes procyonoides*) were recorded, yielding an estimated density of 31.8 ind/km^2^ with a relatively high detection probability (73.7%). In contrast, four point-transect surveys detected 35 water deer (*Hydropotes inermis*), resulting in an estimated density of 30.6 ind/km^2^ but with low detection probability (34.4%) and wide confidence intervals due to limited sample size and complex terrain. These results indicate that real-time drone surveys, when integrated with distance sampling, can effectively correct for detection bias in density estimation of medium- to large-sized mammals. Future studies should apply advanced approaches such as Multiple Covariate Distance Sampling to account for variability in detection probability across survey conditions.

**Supplementary Information:**

The online version contains supplementary material available at 10.1038/s41598-026-50722-9.

## Introduction

 Accurately estimating population density and spatial distribution is a fundamental objective in wildlife ecology and management; however, substantial limitations remain in precisely quantifying population abundance and distribution. Sustained and objective data on fluctuations in animal density and shifts in distribution are crucial for understanding the current state and dynamics of animal communities and for predicting future changes, thereby providing a vital foundation for effective ecosystem management and conservation strategies^1,2^. However, accurately determining wildlife presence and abundance requires substantial survey effort and financial resources, and insufficient or inconsistently collected data can introduce significant uncertainty into management decisions and conservation policy development^3^. These limitations become particularly pronounced in contexts requiring precise population estimates, such as disease management; for example, the European badger (*Meles meles*) in the United Kingdom is recognized as a major reservoir of bovine tuberculosis and has therefore been designated as a target species for intensive research and management^4,5^. However, uncertainty and instability in population estimates continue to hinder the development of effective management policies, including vaccination deployment and population control measures^6^.

Accordingly, a wide range of survey methodologies has been developed and applied to estimate population density and spatial distribution^7–11^. The choice of survey methodology varies according to species-specific ecological traits and behavioral characteristics. For medium- to large-sized mammals, which typically have large home ranges and are difficult to observe directly, sign surveys and camera traps have been widely used^12,13^. Sign surveys are commonly used to estimate population density by accounting for factors such as defecation rates, movement speed, and persistence of signs. In addition, individual identification based on genetic analysis of non-invasive samples such as scats or hair can be used in conjunction with spatial capture–recapture (SCR) models to estimate population density^14^. Camera trap data allow density estimation using capture–recapture (mark–resight) approaches when individual identification is possible. When individuals cannot be reliably identified, alternative approaches such as the Random Encounter Model and camera trap distance sampling have been developed to estimate densities of unmarked animal populations^15–20^. In contrast, for species that are relatively easy to detect directly, population density can be estimated using line-transect–based distance sampling or double sampling approaches^21,22^. However, sign surveys may produce biased estimates due to uncertainty in key parameter estimates, such as average movement speed, defecation rates, and sign persistence^23,24^. In camera trap–based studies, spatial bias can arise when cameras are not placed randomly with respect to animal movement patterns, potentially leading to biased estimates of animal activity or density^25^. Moreover, in rugged or inaccessible terrain, ground-based methods may not only be subject to spatial bias but may also be impossible to implement due to practical reason^26,27^. Consequently, there has been a growing demand for novel approaches that can complement and overcome the limitations of existing survey techniques^28^.

With recent advances in drone technology, novel survey methodologies have been proposed and increasingly applied in wildlife monitoring across a wide range of contexts^29,30^. Drones are small unmanned aerial vehicles capable of surveying large areas within a short time and can take off and land in confined spaces without the need for runways or launch platforms. In addition, drones can extend survey coverage to areas that are difficult to access using conventional methods and, when equipped with camera systems, enable image acquisition from elevated vertical perspectives. Because flight paths can be flexibly designed across diverse terrain, drone surveys may help reduce spatial sampling biases associated with accessibility constraints that often affect ground-based survey methods. Such approaches have been reported to cause relatively less disturbance to wildlife compared with direct human presence^31–34^. Drone-based surveys offer greater cost-effectiveness and operational flexibility compared with conventional manned aerial surveys. For example, the simultaneous operation of multiple drones can reduce survey duration while minimizing duplicate detections resulting from animal movement. Furthermore, image-based observations allow direct verification of animals within survey areas, thereby offering the potential for more precise estimation of population density^35,36^. Although drones have technical limitations related to communication range, recent advances in cellular communication technologies are gradually alleviating these constraints, and their potential applications in animal ecological research are expected to continue expanding.

Drone surveys can be conducted either through post hoc analysis of captured images or video footage, or by identifying animals directly in the field using real-time video feeds from drones^36–39^; more recently, approaches that collect video data across entire survey areas and apply artificial intelligence–based analyses to count individuals have also been actively explored^30,40^. Such image-based automated counting techniques have been primarily applied to surveys of marine mammals and seabirds, where visual obstruction is minimal; however, in forested environments, canopy cover can substantially increase observation error, and surveys relying solely on infrared cameras may further exacerbate uncertainty in species identification^41,42^. In particular, species with similar external morphology may be misidentified, and non-biological objects such as rocks and vegetation can be falsely detected as animals in infrared imagery^29,30^. To address these limitations, real-time drone surveys may help mitigate these challenges in forested environments^43^. This approach typically involves initial detection using infrared cameras, followed by in situ species identification with RGB and zoom cameras when potential targets are detected^29,37^. Such real-time drone surveys can reduce species misidentification by enabling secondary verification through recorded imagery^43^; however, they are subject to operational constraints, as surveyors must manually operate drones in the field while simultaneously making real-time decisions.

When integrated with real-time drone surveys, distance sampling has the potential to effectively address several limitations inherent to real-time drone–based approaches. Although real-time drone surveys allow direct observation of animals in the field and accurate species identification, detection probability can be unstable due to various environmental and operational factors. For example, canopy cover, solar radiation conditions, time of survey, observer experience, and simple human error are major factors influencing animal detection in real-time drone surveys^44,45^. Accordingly, there is a clear need to adopt estimation approaches that can quantitatively evaluate and correct for such detection uncertainty. Distance sampling estimates population density by modeling the decline in detection probability with increasing distance from the survey line or point using a detection function, and by correcting for individuals missed during the observation process based on the estimated function^22^(Supplementary Fig. 1). Distance sampling surveys are commonly implemented using either line-transect or point-transect designs, and the choice of survey design often depends on the characteristics of the study species and the structure of the survey environment. In forested environments, the application of distance sampling has been limited by disturbance caused by observer movement and difficulties in accurately measuring distances to animals; however, these limitations can be largely mitigated by integrating distance sampling with drones, which minimize observer movement and provide a consistent observation altitude. Distance sampling is conceptually well aligned with real-time drone survey approaches, in which observers detect and immediately identify animals in the field; indeed, it has been widely applied in manned aerial surveys^46–48^, but remains only sparsely implemented in drone-based surveys^49,50^. In particular, most previous studies have applied distance sampling to post hoc analyses of recorded imagery, and, to our knowledge, applications integrating distance sampling with real-time drone surveys have not yet been reported. Accordingly, distance sampling in real-time drone survey contexts represents a promising approach for extending density estimation methods that have been well validated in conventional aerial surveys.

The aim of this study was to assess the applicability of distance sampling in real-time drone surveys. To this end, we applied drone-based line-transect and point-transect surveys and used distance sampling to correct for imperfect detection and estimate population density. The surveys were conducted during winter to reduce visual obstruction caused by seasonal vegetation and to improve the detectability of animals in densely vegetated environments. Each survey method was evaluated independently, and the objective of this study was not to compare the relative performance of line- and point-transect approaches. The selection of survey designs was instead primarily determined by the environmental characteristics of each study site.

## Result

### Real-time drone survey detecting

**Fig. 1:**
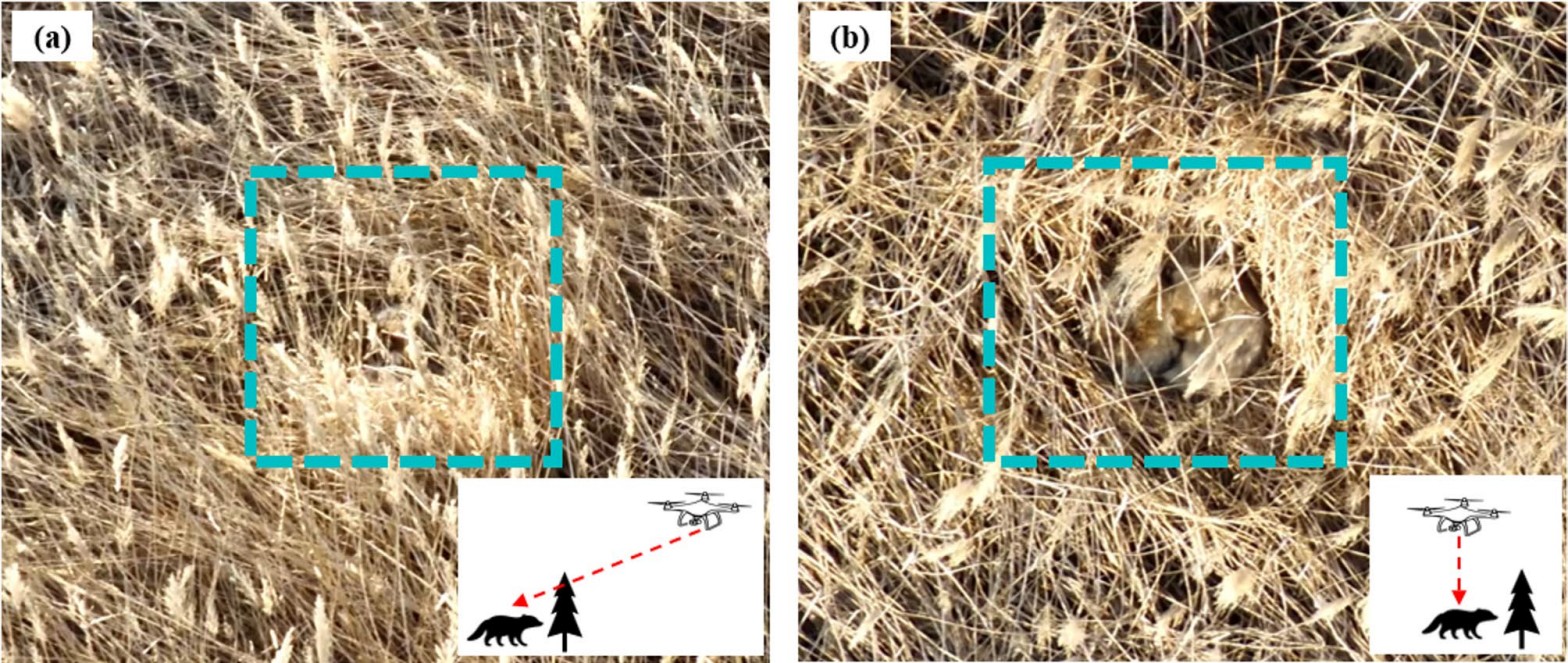
At 08:40 AM on 19 March 2025, the drone’s infrared camera detected a thermal signature presumed to originate from an animal. The automated flight path was interrupted, and the drone was manually navigated to precisely identify the species and count the number of individuals: (**a**) illustrates the animal partially obscured by reeds, complicating the identification of its species and the determination of individual numbers, (**b**) depicts two raccoon dogs clearly identified after the drone was repositioned to hover directly above the target

**Fig. 2:**
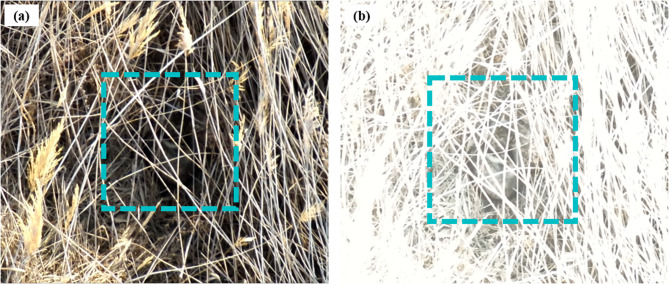
On 25 February 2025, at 8:20 AM, a thermal signal indicative of an animal’s presence was detected by the drone’s infrared camera. The automated flight path was subsequently paused, and manual control of the drone was employed to identify the species and determine the number of individuals: (**a**) depicts a scene in which the animal is concealed beneath reeds, complicating accurate identification of the species and enumeration of individuals, (**b**) displays two raccoon dogs identified following adjustments to the drone camera’s brightness

In the Yeonsu study area, where line-transect surveys were applied, a total of nine drone surveys were conducted, during which 270 raccoon dogs (*Nyctereutes procyonoides*) were observed. In the Chungju study area, where point-transect surveys were applied, a total of four drone surveys were conducted, and 35 water deer (*Hydropotes inermis*) were observed. When the drone was positioned directly above target individuals, both their locations and numbers were clearly identified (Fig. 1). In contrast, individual identification was more challenging in environments with flattened reeds or shaded conditions; however, adjusting image brightness via the drone controller in the field enabled accurate species identification and confirmation of individual counts (Fig. 2). Meanwhile, some targets initially detected as animals were subsequently identified as waste materials or non-target species (Supplementary Fig. 2, 3), indicating that false detections can occur during drone-based detection processes.

### Detection probability estimation from real-time drone surveys using distance sampling

#### Line survey

**Fig. 3:**
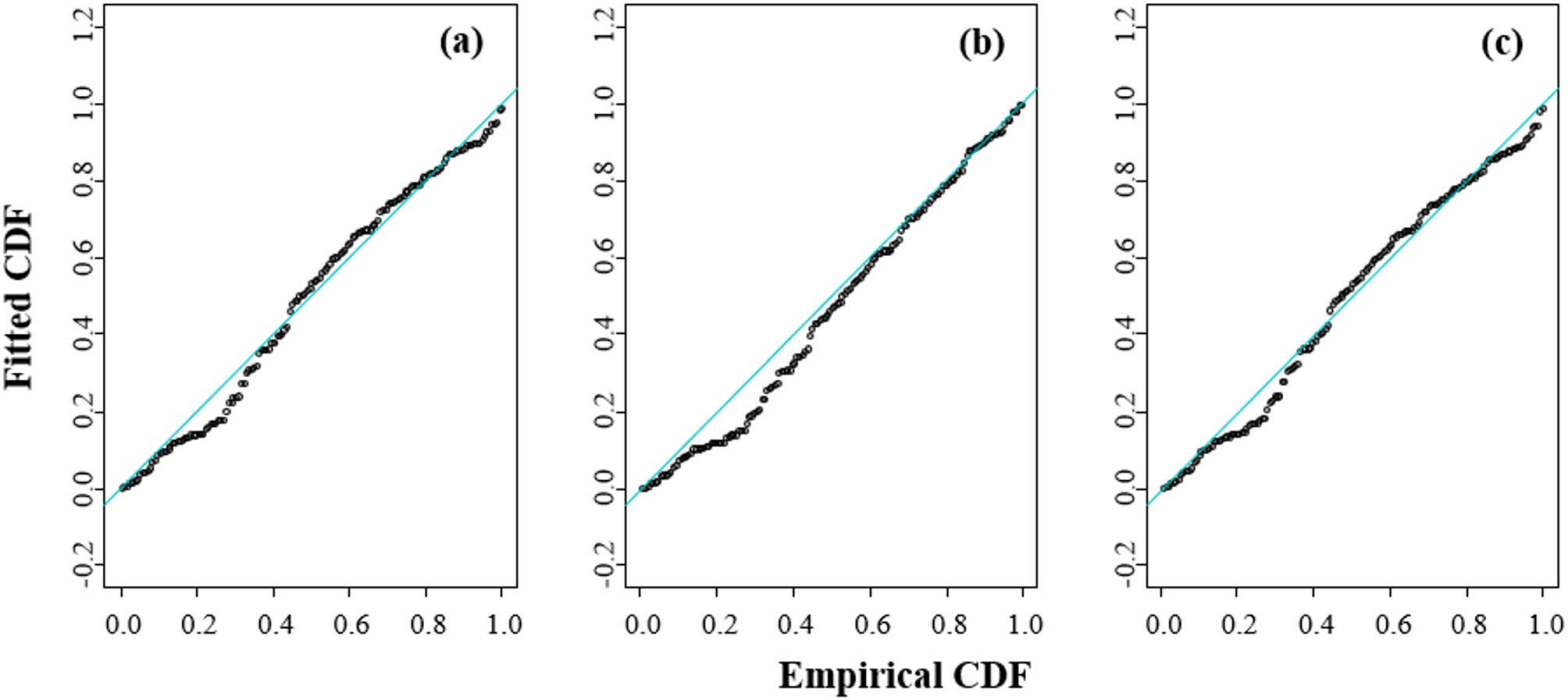
Q–Q plots of distance data for raccoon dogs collected through a line-transect drone survey: (**a**) half-normal model, (**b**) hazard-rate model, (**c**) uniform model with a cosine adjustment, CDF: Cumulative Distribution Function

**Fig. 4:**
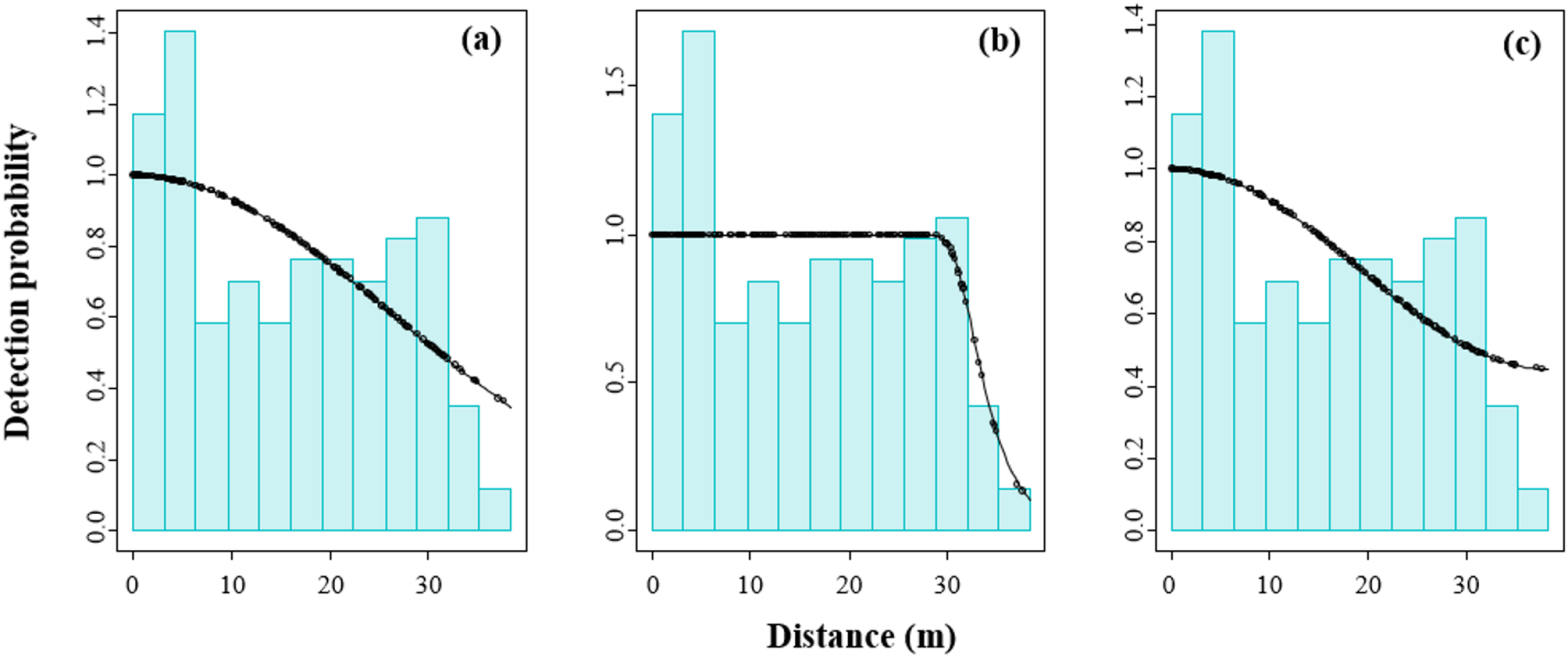
Estimated detection probabilities of raccoon dogs as a function of distance, derived from data obtained through the line-transect drone survey. Panel (**a**) half-normal model, (**b**) hazard-rate model, (**c**) uniform model with cosine adjustment

In the study area where line-transect surveys were applied, a total transect length of 16,322 m was surveyed repeatedly across nine surveys (total effort: 149,091.3 m), resulting in the observation of 152 clusters comprising 270 raccoon dogs. Individuals located beyond the maximum detection distance were excluded from the analysis, resulting in a final dataset comprising 151 clusters and 268 individuals. Model fitting results indicated that the half-normal (HN), hazard-rate (HR), and uniform models with cosine adjustment (UNIF) all showed adequate fit based on both the chi-square and Cramér–von Mises (CvM) goodness-of-fit tests (Table 1). Consistently, quantile–quantile (Q–Q) plots exhibited stable patterns for all three models (Table 1; Fig. 3). Among the three models, the hazard-rate (HR) model yielded the lowest AIC value; however, considering AIC values together with the shape of the estimated detection functions and their agreement with the observed distance histograms, the half-normal (HN) model was selected as the final detection function (Table 1; Fig. 4). The detection probability estimated from the HN model was 73.7%, and the resulting density estimate was slightly higher than the naïve density (ND) estimate (Table 1).

**Table 1:** Detection rates, density estimates, and goodness-of-fit statistics for distance sampling models derived from real-time drone line-transect surveys. ND: Naive Density, HN: half-normal model, HR: hazard-rate model, UNIF: uniform with cosine adjustment model.

Model	AIC	Detection rate (%)	Density (clusters/km^2^) [95% CI]	Density (individuals/km^2^) [95% CI]	Chi-square tests *p*-value	CvM Test *p*-value
ND			13.2	23.4		
HN	1091.0	73.7	17.9 [13.7–23.4]	31.8 [25.7–39.2]	0.09	0.31
HR	1089.3	88.5	14.9 [8.4–26.4]	26.4 [18.9–37.1]	0.25	0.10
UNIF	1093.0	72.4	18.2 [14.0–23.7]	32.3 [18.9–37.1]	0.06	0.33

#### Point-transect survey

**Fig. 5:**
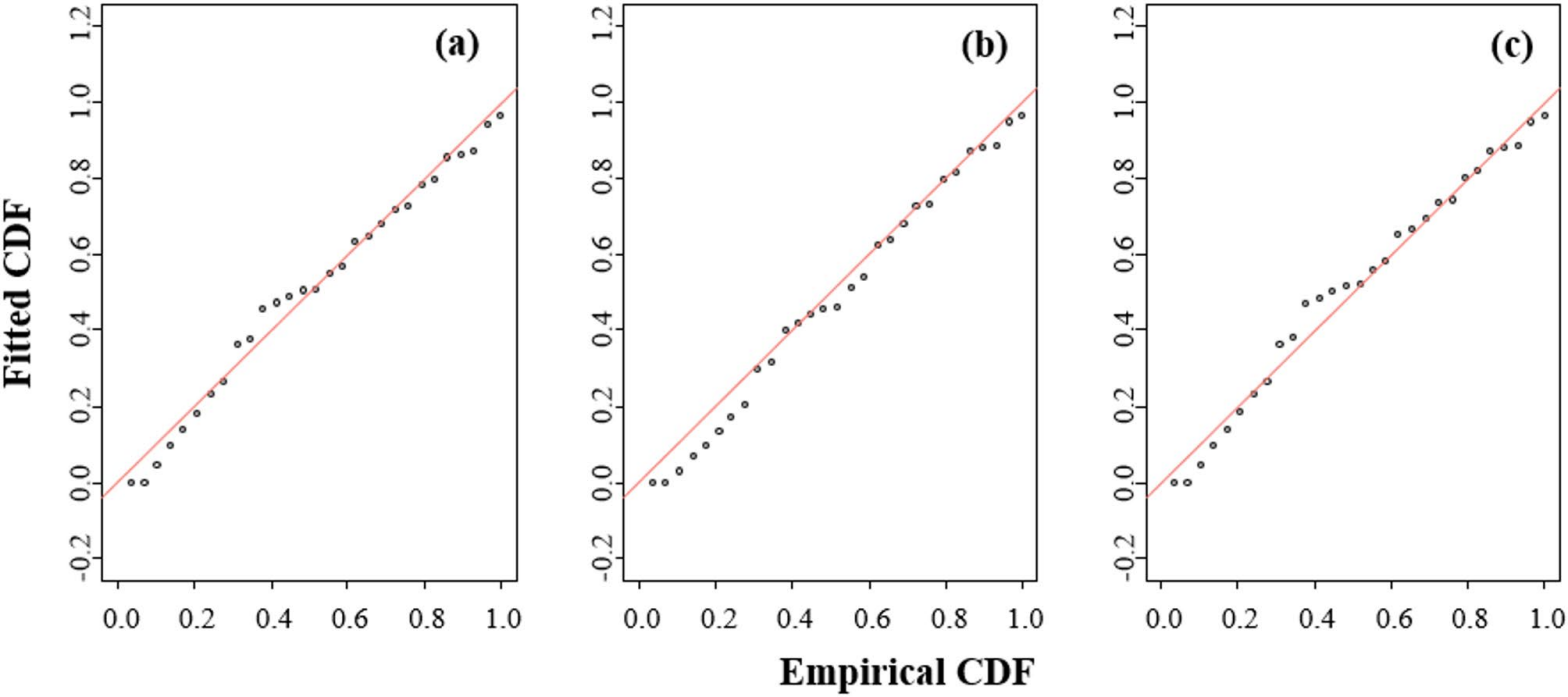
Q–Q plots of distance data for water deer collected through a point-transect drone survey: (**a**) half-normal model, (**b**) hazard-rate model, (**c**) uniform model with a cosine adjustment, CDF: Cumulative Distribution Function

**Fig. 6:**
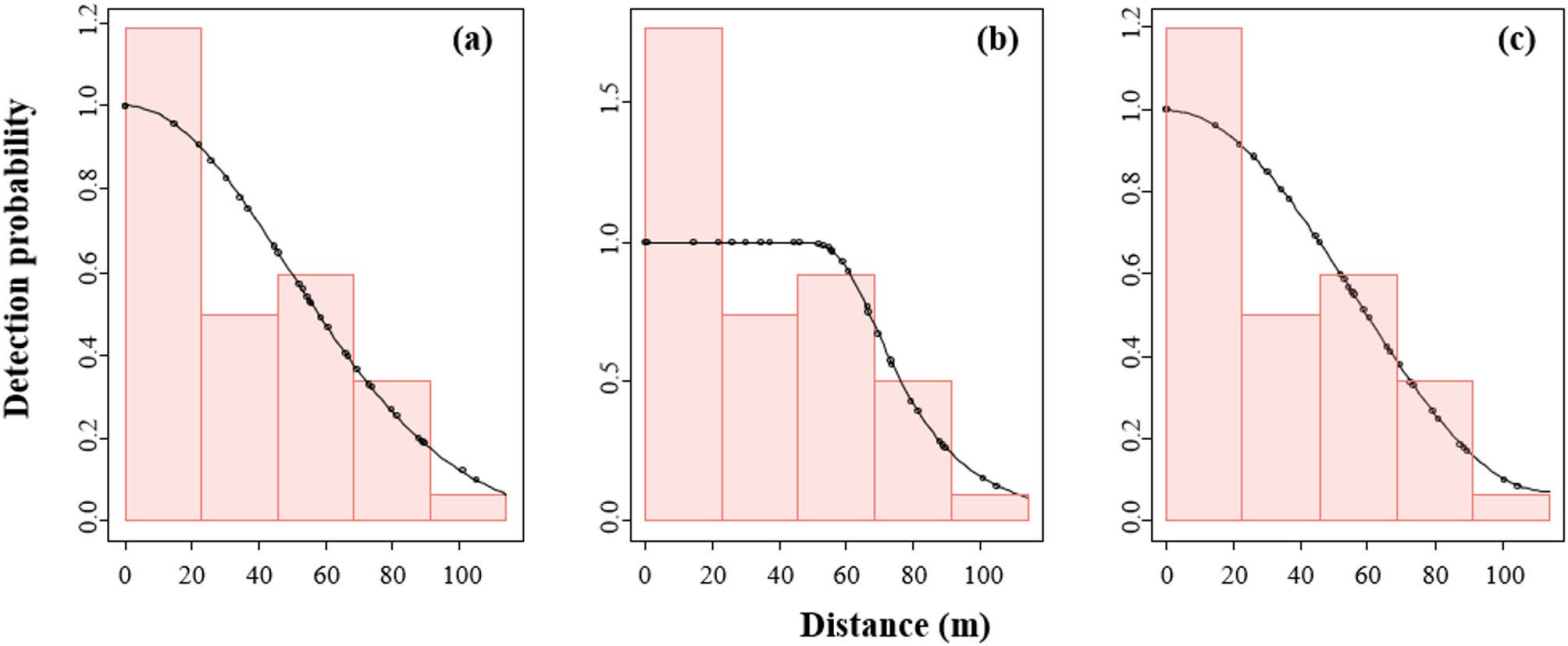
Estimated detection probabilities of water deers as a function of distance, derived from data obtained through the point-transect drone survey: (**a**) half-normal model, (**b**) hazard-rate model, (**c**) uniform model with cosine adjustment.

In the point-transect surveys, four repeated surveys were conducted across 19 points (totaling 76 points surveys), resulting in the detection of 31 clusters comprising 35 water deer. Detections located beyond the maximum detection distance were excluded, resulting in a final dataset comprising 29 clusters and 33 individuals. Model fitting results indicated that the half-normal (HN), hazard-rate (HR), and uniform with cosine adjustment (UNIF) models all showed adequate fit based on both the chi-square and Cramér–von Mises (CvM) goodness-of-fit tests (Table 2). Consistently, Q–Q plots exhibited stable patterns across all three models (Table 2; Fig. 5). However, all three models yielded wide confidence intervals for the estimated densities, indicating substantial uncertainty in density estimation (Table 2). Among the three models, the half-normal (HN) model exhibited the lowest AIC value and was selected as the final detection function after jointly considering the shape of the estimated detection function and its agreement with the observed distance histogram (Table 2; Fig. 6). Under the half-normal (HN) model, the estimated detection probability was low (34.4%), and the corresponding density estimate was higher than the naïve density (ND) (Tables 2, 3, and 4).

**Table 2:** Detection rates, density estimates, and goodness-of-fit statistics for distance sampling models derived from real-time drone point-transect surveys. ND: Naive Density, HN: half-normal model, HR: hazard-rate model, UNIF: uniform with cosine adjustment model.

Model	AIC	Detection Rate (%)	Density (Clusters/km^2^) [95% CI]	Density (Individuals/km^2^) [95% CI]	Chi-square tests *p*-value	CvM Test *p*-value
ND			9.3	10.6		
HN	289.0	34.4	27.1 [15.7–46.9]	30.6 [17.9–52.5]	0.46	0.93
HR	291.1	51.1	18.2 [10.7–31.1]	20.6 [12.3–34.4]	0.31	0.97
UNIF	289.1	34.7	26.9 [17.6 − 41.1]	30.3 [20.2–45.5]	0.50	0.86

**Table 3:** Detection rates, density estimates, and goodness-of-fit statistics for distance sampling models derived from real-time drone line-transect surveys. ND: Naive Density, HN: half-normal model, HR: hazard-rate model, UNIF: uniform with cosine adjustment model.

Model	AIC	Detection Rate (%)	Density (Clusters/km^2^) [95% CI]	Density (Individuals/km^2^) [95% CI]	Chi-square tests *p*-value	CvM Test *p*-value
ND			13.2	23.4		
HN	1091.0	73.7	17.9 [13.7–23.4]	31.8 [25.7–39.2]	0.09	0.31
HR	1089.3	88.5	14.9 [8.4–26.4]	26.4 [18.9–37.1]	0.25	0.10
UNIF	1093.0	72.4	18.2 [14.0–23.7]	32.3 [18.9–37.1]	0.06	0.33

**Table 4:** Detection rates, density estimates, and goodness-of-fit statistics for distance sampling models derived from real-time drone point-transect surveys. ND: Naive Density, HN: half-normal model, HR: hazard-rate model, UNIF: uniform with cosine adjustment model.

Model	AIC	Detection Rate (%)	Density (Clusters/km^2^) [95% CI]	Density (Individuals/km^2^) [95% CI]	Chi-square tests *p*-value	CvM Test *p*-value
ND			9.3	10.6		
HN	289.0	34.4	27.1 [15.7–46.9]	30.6 [17.9–52.5]	0.46	0.93
HR	291.1	51.1	18.2 [10.7–31.1]	20.6 [12.3–34.4]	0.31	0.97
UNIF	289.1	34.7	26.9 [17.6 − 41.1]	30.3 [20.2–45.5]	0.50	0.86

## Discussion

In this study, we evaluated the applicability of distance sampling in real-time drone surveys through independent case studies conducted under different environmental conditions. Rather than directly comparing survey methods, the aim of this study was to examine whether distance sampling can be effectively integrated with real-time drone observations to estimate wildlife density under field conditions. Our results demonstrate that real-time drone surveys combined with distance sampling can provide quantitative estimates of wildlife density, even in environments where conventional ground-based surveys are logistically constrained or impractical. Importantly, these findings should be interpreted within the specific ecological contexts of each study site, as both species characteristics and habitat conditions strongly influence detection processes and resulting density estimates.

The estimated densities of raccoon dogs in the Yeonsu area of Incheon and water deer in the Chungju area were higher than previously reported nationwide averages^51^. However, these comparisons should be interpreted with caution, as previously reported values typically represent broad-scale estimates, whereas the present study provides local density estimates derived from specific habitats intensively used by the target species. For raccoon dogs, local aggregation may have occurred due to abundant food resources and limited available habitat in urban environments, while water deer are known to prefer hilly terrain with access to water resources^52,53^. Given that the study sites consisted of habitat types favorable to each species, these environmental characteristics likely contributed to the elevated local density estimates. These results indicate that wildlife density is strongly influenced by habitat-specific conditions and resource availability, and therefore extrapolation from localized surveys to broader spatial scales requires careful consideration of habitat heterogeneity.

A key finding of this study is that distance sampling can be effectively applied to real-time drone surveys under appropriate conditions to account for imperfect detection and to improve the reliability of density estimation. In our results, detection probability decreased with increasing distance from the center of the camera frame, likely due to visual obstruction and observer-related biases during real-time monitoring. Individuals located near the periphery of the camera frame were more likely to be obscured by vegetation^54^, and central fixation bias may have further influenced detection patterns^55^. These characteristics suggest that simple count-based approaches may lead to biased estimates if detection probability is not explicitly considered. By incorporating detection functions, distance sampling provides a framework for correcting such biases and enables more robust estimation of wildlife density. In this study, real-time drone surveys generally satisfied key assumptions required for distance sampling, including accurate distance measurement and high detectability of individuals located near survey lines or points.

In addition to these methodological aspects, drone-based surveys provide practical advantages that facilitate the application of distance sampling, particularly in environments where conventional survey methods are difficult to implement. In densely vegetated habitats such as reedbeds, ground-based surveys are often constrained by limited accessibility, reduced visibility, and difficulties in deploying camera traps or conducting sign-based surveys^56^. For example, the raccoon dog survey conducted in reedbed habitats represents a case where conventional methods are largely infeasible due to swampy ground conditions and dense vegetation structure. In contrast, real-time drone surveys allow direct observation of animals from above the vegetation layer, enabling data collection in otherwise inaccessible environments. Furthermore, drones enable relatively rapid coverage of survey areas, which may help approximate the temporal snapshot conditions assumed in distance sampling. These characteristics suggest that drone-based surveys are well suited for the application of distance sampling, as they facilitate the satisfaction of key assumptions, including accurate distance measurement, high detectability near the survey line or point, and the ability to approximate snapshot survey conditions^57^.

Despite these advantages, several limitations should be considered when interpreting the findings of this study. First, the analyses were based on independent case studies conducted under different species and environmental conditions, and therefore the results should be interpreted as case-specific rather than broadly generalizable. In particular, the surveys were not conducted under a unified design within a single study area, which limits the ability to isolate methodological effects and precludes direct comparison between survey approaches. Second, the point-transect case study exhibited higher uncertainty, which may be associated with both a relatively small sample size and terrain complexity. A limited number of detections can reduce the stability of detection function estimation, while complex topography may affect the accuracy of distance measurements, particularly when perpendicular distance does not adequately represent true spatial distance in non-flat environments^58^. These factors suggest that point-transect applications of distance sampling in drone surveys may be sensitive to both sample size and terrain conditions. Finally, detection probability is influenced by multiple environmental and observational factors, including vegetation structure, climatic conditions, and survey timing^44,45,59^. Because this study was conducted under relatively specific conditions, variability across broader environmental gradients was not fully captured. Future studies incorporating larger datasets across diverse habitats, as well as approaches such as multiple covariate distance sampling (MCDS), would improve the robustness and broader applicability of this framework.

In conclusion, this study provides case-based evidence that distance sampling can be effectively integrated with real-time drone surveys to estimate wildlife density while accounting for imperfect detection. The primary contribution of this study lies in demonstrating the applicability of distance sampling within drone-based survey frameworks, rather than in evaluating survey methods or promoting drone technology alone. While the applicability of this approach depends on species, habitat conditions, and survey design, the integration of drone observations with distance sampling offers a promising methodological approach for wildlife monitoring, particularly in environments where conventional survey methods are limited or impractical.

## Method

### Study area

**Fig. 7:**
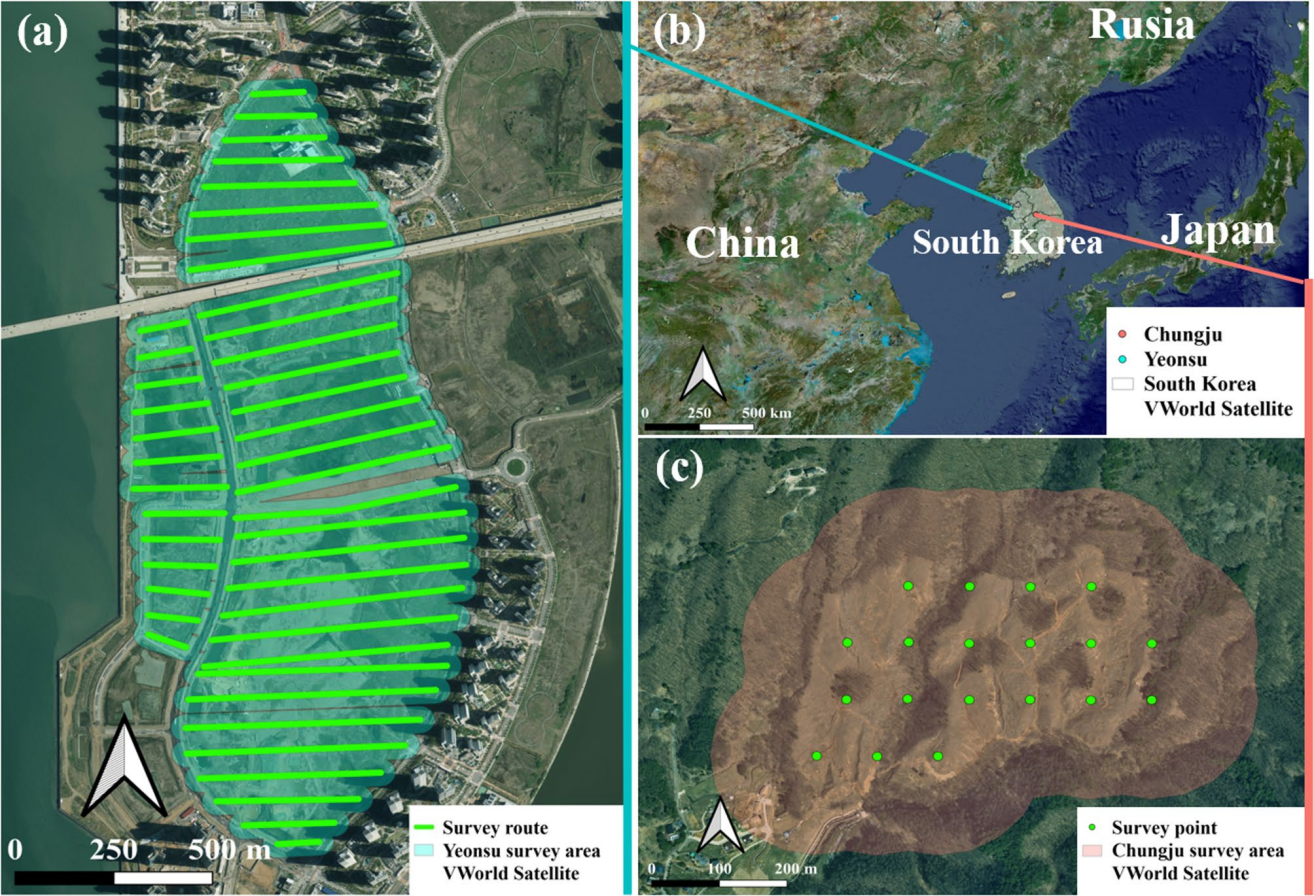
Locations of study sites. (**a**) line-transect survey routes in Yeonsu, (**b**) An overview of South Korea highlighting the two survey regions, and (**c**) point-transect survey routes in Chungju. Satellite imagery was obtained from VWorld (https://www.vworld.kr), and the map was generated using QGIS (version 3.40.7; https://www.qgis.org).

All study areas were located in the Republic of Korea (South Korea) (Fig. 7). The Korean Peninsula lies within the mid-latitude temperate climate zone and is characterized by four distinct seasons: spring, summer, autumn, and winter. During the winter season, when this study was conducted, climatic conditions are generally influenced by cold and dry continental high-pressure systems, resulting in low temperatures and limited precipitation.

#### Yeonsu study site

The line-transect survey site (37°24′7.50″N, 126°36′57.96″E) was located in Yeonsu-gu, Incheon, Republic of Korea (Fig. 7). The Yeonsu study area is part of an international new town where land reclamation has been ongoing since 1994. The region has a mean annual temperature of 12.5 °C, with a minimum temperature of − 1.5 °C and a maximum temperature of 25.6 °C. Mean annual precipitation is 1,207.4 mm, and mean annual relative humidity is 68.8%. Some undeveloped areas remaining after reclamation consist primarily of salt marshes and extensive reedbeds, which provide habitat for a limited number of mammal species, including raccoon dog, weasel, and feral cat. The total area of the Yeonsu study site used in this study was 1.03 km^2^.

#### Chungju study site

The point-transect survey site (37°6′33.77″N, 127°56′43.20″E) was located in Chungju, Republic of Korea (Fig. 7). The study area is situated at elevations ranging from 151 to 220 m above sea level and is characterized by gently sloping terrain (5–21°) with predominantly southwest-facing aspects. Chungju has a mean annual temperature of 11.7 °C, with a minimum temperature of − 3.2 °C and a maximum temperature of 25.2 °C. Mean annual precipitation is 1,214.3 mm, and mean annual relative humidity is 67.3%. The point-transect study area is dominated by deciduous forest. In 2023, retained harvesting was conducted over approximately 0.15 km^2^, and the area is currently in a semi-open forest condition undergoing thinning operations. The site supports a diverse assemblage of medium- to large-sized mammals, including wild boar (*Sus scrofa*), water deer, roe deer (*Capreolus pygargus*), badger (*Meles leucurus*), raccoon dog, and leopard cat (*Prionailurus bengalensis*). The total area of the Chungju study site was 0.15 km^2^.

### Data collection

**Fig. 8:**
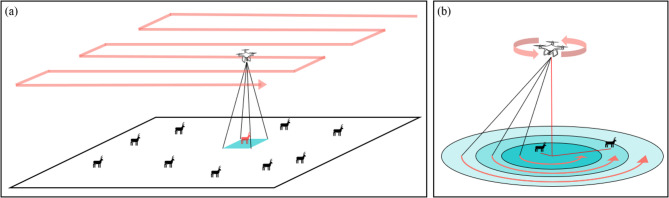
Drone survey methods: (**a**) line survey method and (**b**) point survey method.

All drone surveys were conducted as real-time drone surveys using a Matrice 30T (DJI, China). The Matrice 30T is a compact unmanned aerial vehicle with a maximum takeoff weight of 4.1 kg and dimensions of 470 × 858 × 215 mm, and it is equipped with an omnidirectional obstacle sensing system. The payload includes an integrated camera system comprising infrared (IR), RGB (visible light), and zoom cameras. The maximum transmission range of the remote controller is approximately 15 km under interference-free conditions; however, in urban environments with strong electromagnetic interference, the effective range is typically reduced to approximately 1.5–3 km. Although the manufacturer-specified maximum flight time per battery is approximately 41 min, survey design in this study was based on a conservative maximum flight time of ~ 25 min to account for reduced battery efficiency associated with terrain and survey conditions. Surveys were conducted by initially detecting animals using the infrared (IR) camera. When a potential animal target was identified, the automated flight mission was temporarily paused, and species identification was performed using the RGB zoom camera. The thermal sensor has a resolution of 640 × 512 pixels with a temperature accuracy of ± 2 °C, and the zoom camera is equipped with a 48-megapixel sensor. When RTK (Real-Time Kinematic) positioning was enabled, the horizontal and vertical positional accuracies of both sensors were approximately ± 1 cm and ± 1.5 cm, respectively. After species identification, the precise geographic coordinates of each detected individual were recorded using the onboard laser rangefinder and target localization functions. Because infrared-based drone surveys are strongly influenced by solar radiation conditions, surveys were conducted either from sunrise to within 3 h after sunrise or from 3 h before sunset until sunset. To monitor raccoon dogs and water deer, real-time drone surveys were implemented using both line-transect and point-transect designs^29^(Fig. 8). When an object presumed to be an animal was detected during a survey, automated flight was paused to allow for species identification. If species identification was uncertain due to image resolution or viewing conditions, additional confirmation was conducted by manually adjusting the drone position or modifying image brightness settings. To minimize disturbance to animals caused by drone movement, flight altitude was not reduced below 50 m during surveys^29^. Although drone-based studies targeting medium- to large-sized mammals in Korea remain limited, previous research using thermal imaging drones has suggested that flight altitudes around 80 m can provide an effective balance between detection performance, survey coverage, and operational safety^60^. Based on these findings, and considering the sensor capability and operational conditions of the Matrice 30T, survey altitudes of approximately 70–80 m were adopted in this study. Nevertheless, optimal flight altitude may vary depending on species body size, habitat structure, and environmental conditions, and therefore practical field conditions and operator experience were also considered during survey implementation.

### Line-transect survey

The line-transect survey was conducted in the Yeonsu study area, which consists of relatively flat terrain with extensive reed habitats that allow systematic flight paths across the landscape. Under such conditions, line-transect surveys provide efficient spatial coverage and were therefore adopted as the survey design for this site. The survey was conducted from February to April 2025, during winter when vegetation cover was minimal and visibility was maximized. The target species for this survey was the raccoon dog, a medium- to small-sized carnivorous mammal. Raccoon dogs were selected as the focal species because they were among the most frequently observed medium-sized mammals in the Yeonsu study area during drone surveys. Surveys were performed by flying the drone along predefined waypoints at an altitude of 80 m and a flight speed of 7 m/s, with the camera fixed at a nadir angle (90°) to observe the ground surface directly. A total of 44 straight transect lines were systematically arranged at regular intervals across the study area. The spacing between adjacent transects was set to 80 m to minimize overlap between survey paths. However, due to spatial constraints within the study area, partial overlap between transects was unavoidable in some sections.

### Point-transect survey

The point-transect survey was conducted in the Chungju study area, where forested habitats and sloped terrain limit the feasibility of systematic line-transect flights. Under such conditions, point-based observations provide a practical survey framework for detecting animals from fixed aerial positions. The survey was conducted from December 2024 to March 2025, a period when leaf fall in forested areas improves ground visibility. The target species for this survey was the water deer, a medium-sized ungulate. Water deer were selected as the focal species because they were one of the most frequently observed medium-sized mammals in the Chungju study area during drone surveys. A total of 19 survey points were established, and surveys at each point were conducted at an altitude of 70 m. Under flat terrain conditions, each survey point covered an effective survey radius of approximately 79 m. The distance between adjacent survey points was set to 90 m, resulting in partial overlap among survey areas. During point-transect surveys, observations were conducted by sequentially adjusting the camera angle to 6°, 15°, and 30°. At each angle, the drone completed one full rotation, resulting in a total of three rotational observations per survey point.

### Data analyses

In this study, wildlife detection and density estimation were conducted using real-time drone surveys. Density was estimated by applying distance sampling methods to drone survey data. To improve estimation accuracy, repeated surveys were conducted within the same study areas to ensure sufficient sample sizes (nine line-transect surveys in Yeonsu and four point-transect surveys in Chungju). Collected data were analyzed using a pooling approach, in which survey effort and observed individuals from all survey replicates were combined to estimate a single detection function. Three distance sampling models—the half-normal model (HN), hazard-rate model (HR), and uniform model with cosine adjustment (UNIF) were fitted to the real-time drone survey data, and model performance was evaluated to identify the most appropriate detection function. Final model selection was based not only on comparisons of Akaike’s Information Criterion (AIC) values but also on visual assessments of the agreement between the estimated detection functions and the observed distance histograms^61^. In addition to AIC-based model comparison, absolute model fit was evaluated using goodness-of-fit diagnostics. Specifically, Cramér–von Mises and chi-square goodness-of-fit tests were applied, and quantile–quantile (Q–Q) plots were examined to assess the agreement between the empirical cumulative distribution function of observed distances and the cumulative distribution function fitted by each detection model. For comparison with distance sampling–based estimates, a naïve density (ND) was also calculated by dividing the number of observed individuals by the surveyed area. When calculating ND, the surveyed area was defined as the effective survey area derived from distance sampling rather than the total study area. Specifically, the effective survey area was calculated as effort × 2w for line-transect surveys and effort × πw^2^ for point-transect surveys, where effort represents the total transect length or the number of survey points, and w denotes the truncation distance. For the DJI Matrice 30T equipped with an infrared camera, the horizontal field of view at an altitude of 80 m corresponds to an approximate swath width of 76.8 m; thus, the truncation distance for the line-transect survey was set to 38.4 m. For the point-transect survey, the truncation distance was determined as the 95th percentile of the observed distance distribution to account for variable observation conditions caused by terrain slope, resulting in a truncation distance of approximately 114 m. All density estimations and statistical analyses were performed using R version 4.5.0. Detection probabilities and density estimates were obtained using the Distance package in R^62^.

## Supplementary Information

Below is the link to the electronic supplementary material.

Supplementary Material 1

## Data Availability

The dataset is available from the corresponding authors upon request.
