# Supplementary material for: Estimating the density of raccoon dogs (Nyctereutes procyonoides) and water deer (Hydropotes inermis) using thermal real-time drone surveys and distance sampling
Source: Sci Rep. 2026 Apr 28;16:21941. doi: 10.1038/s41598-026-50722-9 (PMC13365454; doi:10.1038/s41598-026-50722-9)
Supplement: Supplementary file 1 — Supplementary Material 1 [file 41598_2026_50722_MOESM1_ESM.docx]

Supplementary


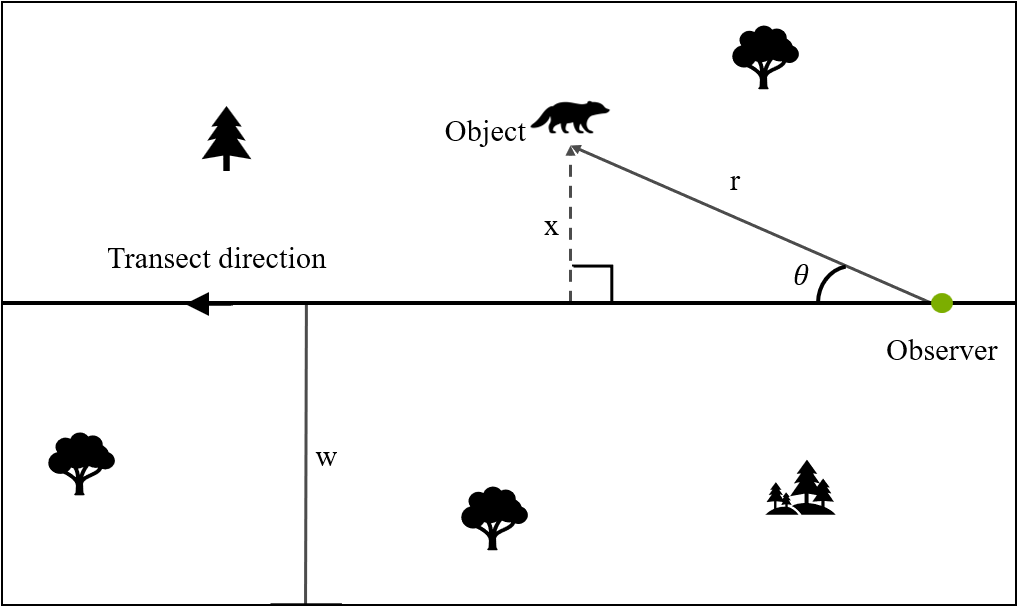


Supplementary Fig. 1. Schematic illustration of the line transect distance sampling method. An observer moves along a predefined transect line and records detections of objects (e.g., animals). For each detection, the perpendicular distance (x) from the transect line is calculated based on the radial distance (r) and the detection angle (θ). W denotes the truncation distance, representing the maximum perpendicular distance considered in the analysis.


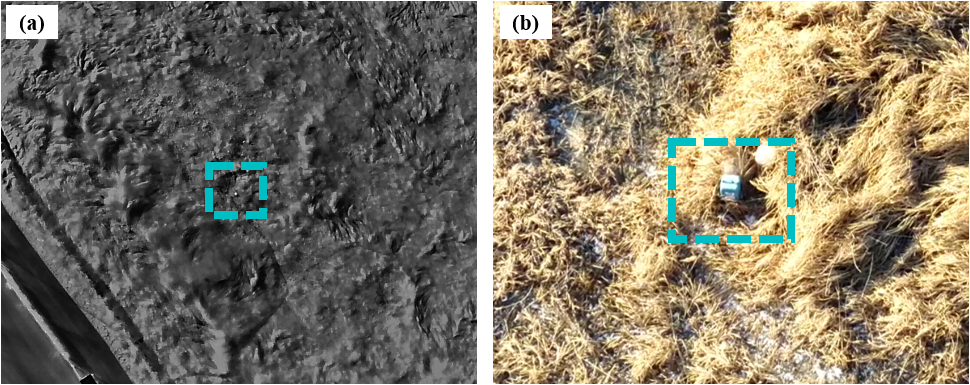


Supplementary Fig. 2. On 13 February 2025 at 08:55 AM, a non-living object was erroneously identified as an animal: (a) depicts a thermal signal recorded by the infrared camera, (b) displays the same object, identified as trash upon detailed examination with the zoom camera.


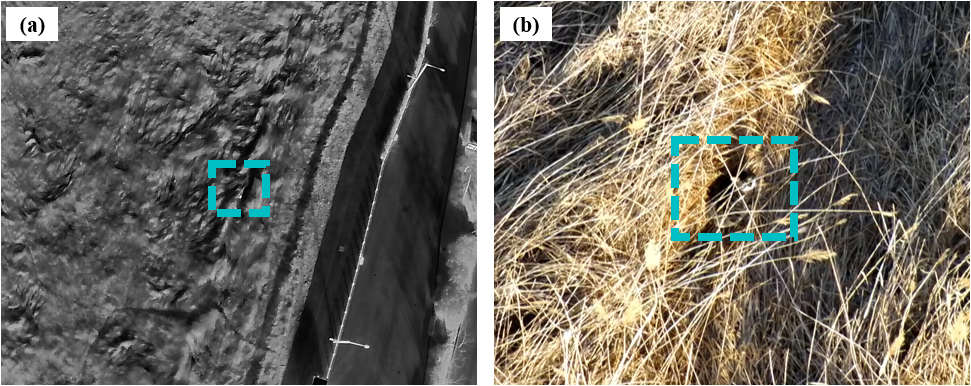


Supplementary Fig. 3. On 13 February 2025 at 08:56 AM, an example of a non-target species being misidentified as the target species: (a) shows a thermal signal captured by the infrared camera, (b) presents the same individual later identified as a cat upon inspection with the zoom camera.


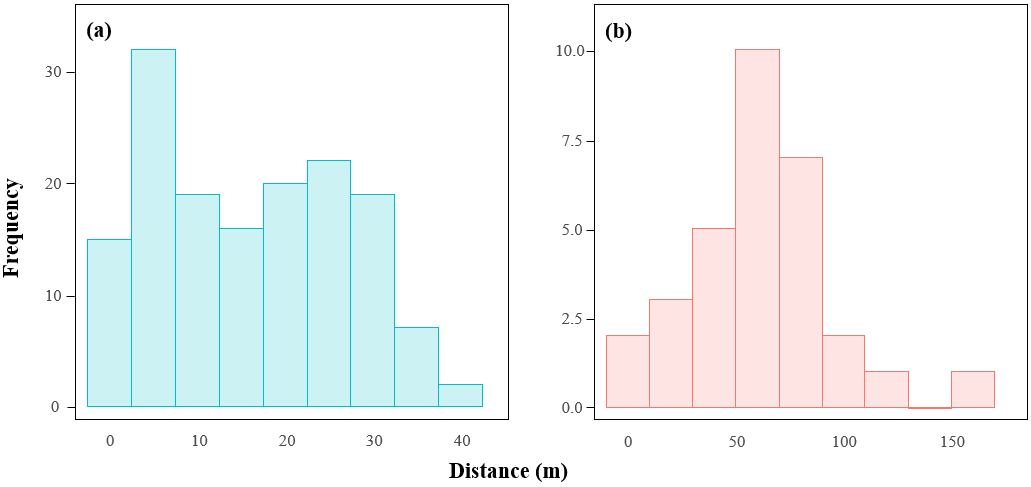


Supplementary Fig. 4. Distributions of the distances to detected animals. Panel (a) shows the results of a line-transect drone survey targeting raccoon dogs, (b) presents the results of a point-transect drone survey targeting water deer.
